# Phenotype Accessibility and Noise in Random Threshold Gene Regulatory Networks

**DOI:** 10.1371/journal.pone.0119972

**Published:** 2015-04-28

**Authors:** Ricardo Pinho, Victor Garcia, Marcus W. Feldman

**Affiliations:** 1 Department of Biological Sciences, Stanford University, Stanford, California, USA; 2 PhD Program in Computational Biology, Instituto Gulbenkian de Ciência, Oeiras, Portugal; 3 Institute of Integrative Biology, ETH Zurich, Zurich, Switzerland; Institute of Molecular and Cell Biology, SINGAPORE

## Abstract

Evolution requires phenotypic variation in a population of organisms for selection to function. Gene regulatory processes involved in organismal development affect the phenotypic diversity of organisms. Since only a fraction of all possible phenotypes are predicted to be accessed by the end of development, organisms may evolve strategies to use environmental cues and noise-like fluctuations to produce additional phenotypic diversity, and hence to enhance the speed of adaptation. We used a generic model of organismal development --gene regulatory networks-- to investigate how different levels of noise on gene expression states (i.e. phenotypes) may affect access to new, unique phenotypes, thereby affecting phenotypic diversity. We studied additional strategies that organisms might adopt to attain larger phenotypic diversity: either by augmenting their genome or the number of gene expression states. This was done for different types of gene regulatory networks that allow for distinct levels of regulatory influence on gene expression or are more likely to give rise to stable phenotypes. We found that if gene expression is binary, increasing noise levels generally decreases phenotype accessibility for all network types studied. If more gene expression states are considered, noise can moderately enhance the speed of discovery if three or four gene expression states are allowed, and if there are enough distinct regulatory networks in the population. These results were independent of the network types analyzed, and were robust to different implementations of noise. Hence, for noise to increase the number of accessible phenotypes in gene regulatory networks, very specific conditions need to be satisfied. If the number of distinct regulatory networks involved in organismal development is large enough, and the acquisition of more genes or fine tuning of their expression states proves costly to the organism, noise can be useful in allowing access to more unique phenotypes.

## Introduction

Development processes are key in mapping genotypes to phenotypes, often by complex and poorly understood regulatory mechanisms [1, 2]. The nonlinearity of the genotype-phenotype map can induce considerable *degeneracy* [3], such that many genotypes develop the same phenotype while simultaneously rendering some potential phenotypes inaccessible to evolutionary search [4]. The developmental process might therefore entail a reduction of the phenotypic variation in these organisms to a fraction of all potential variability, which constrains the evolutionary pathways that populations or species can take [4, 5].

A large body of theoretical research has shown that developmental constraints are of great significance for the study of evolution [6–9]. Intriguingly, some shortcomings of reduced phenotypic variation have been shown to be partially compensated for by noise in gene expression [10, 11].

Noise is ubiquitous in gene expression processes [12, 13], and organisms have evolved different mechanisms to adapt to it [12, 14]. Some organisms employ mechanisms that use noise to their advantage, making it essential to gene expression [15, 16]. For example, small fluctuations in protein concentration can lead to significant changes in cell fate, and allow isogenic cells to develop different phenotypes, thus increasing phenotypic variability [13, 17]. Besides phenotypic variability, noise in gene expression has been associated with coordinating the expression of a large set of genes, stochastic phenotype switching, and facilitating evolutionary adaptation [15, 16]. Conversely, very high levels of noise might have detrimental, destabilizing effects on development, adversely affecting a population’s diversity [12].

To address these issues in a widely studied model of development, we studied how noise affects access to new phenotypes that are inaccessible by deterministic gene networks. To this end, we focused on a particular class of random Boolean networks known as Random Threshold Networks [18]. These networks act on initial phenotypes and produce the subsequent changes that define the course of development, until an end state or fixed point is reached, or alternatively some other behavior results. We investigated the effects of noise on two of the routes by which organisms may increase their phenotypic variability, namely by acquiring more genes or by adopting more gene expression states.

We studied different network model types, namely *binary networks*, i. e. networks that only allow for up- or down-regulation on genes, and *real-valued networks*, which can exert a regulatory signal of arbitrary strength. Both types of networks were studied in settings where they could be randomly chosen from the set of all possible networks (termed *random networks*) or alternatively, where they were pre-selected for the stability of their phenotypic outcomes (termed *stable networks*). Additionally, we generalized some aspects of the Boolean framework by allowing multiple gene expression states for the phenotype (not exclusively “on” or “off”).

We found that standard binary network models with two gene expression states can access the maximum number of unique phenotypes by development if enough random networks are sampled, but not if more gene expression states are considered. In general, irrespective of the noise level, stable networks access more phenotypes if only two gene states can be expressed, but less phenotypes if more are expressed.

In general, noise decreases phenotype discovery. However, in developmental models that allow for more expression states, noise can enhance the discovery of new phenotypes, but only under very specific conditions and to a very moderate extent. Real-valued networks strongly enhance phenotype discovery, but again this is negatively affected by noise. These results are robust to the different implementations of noise investigated.

## Methods

To study how distinct gene expression or phenotype states are attained during development, we implemented an extensively studied model of gene network dynamics [4, 19–25]. The model comprises four components: (1) A gene interaction network that regulates (2) the gene expression state by means of (3) a normalization function. The expression state is subject to (4) noise.

### Interaction Matrix

The interaction network consists of *N* genes, represented by an *N* × *N* matrix, *W*, whose elements, *w*_*ij*_, denote the effect on gene *i* of the product of gene *j*. We think of the matrix *W* as representing a genotype, which, in concert with other factors, will codetermine the end state of the gene expression. The matrix is not symmetric and diagonal elements, *w*_*ii*_, represent autoregulation.

### Gene expression states

Gene expression is represented by a state vector *S*(*t*) = (*s*_1_(*t*), …, *s*_*N*_(*t*)). The values of *s*_*i*_ represent the expression levels of gene *i* at time *t*. To investigate one possibility for augmentation of the phenotype space open to organisms, we generalized the commonly utilized binary entry values *s*_*i*_ ∈ {−1, 1}, usually restricted to two states [19, 21], to *k* gene expression states, evenly spaced over the interval [0, 1]. The off state of a gene, *s*_off_, is often represented by −1 [19, 21]. We choose the more realistic *s*_off_ = 0, since an expression state of −1 will affect the expression of other genes [23, 24, 26]. With *s*_off_ = 0, *s*_*i*_ can take values given by

$$\begin{array}{c} s_{i}=\frac{j}{k-1},j=0,1,\cdots ,k-1. \end{array}$$ (1)

Standard techniques for the simulation of gene regulatory networks involve either binary gene expression states or a continuum of gene expression levels. On the one hand, the notion that genes are either expressed or not is a useful simplification for the computational investigation of gene regulatory networks, but lacks realism. On the other hand, the idea that all possible gene expression levels can be attained with infinite precision is equally unrealistic, since transcription factor proteins are produced in discrete numbers and are likely to exert different regulatory strengths when binding to promoter regions. Considering discrete levels of gene expression, as we propose by using different *k*, is therefore a more realistic compromise between these two extremes.

### Dynamics

The deterministic, discrete-time dynamics of *S*(*t*) is modeled by the set of nonlinear coupled difference equations

$$\begin{array}{c} s_{i}\left(t+1\right)=f\;(\sum_{j=1}^{N}w_{ij}\left(s_{j}\left(t\right)+\epsilon \right)), \end{array}$$ (2)

where *f* is a normalization function that prevents the system from diverging and *ϵ* is drawn from the normal distribution 𝓝(0, *σ* ^2^), *σ* = 0, 0.01, 0.05, …, 0.25. If not stated otherwise, the noise term, *ϵ* is added at each time step *t* and independently for each gene, as in [24, 27].

The Gaussian noise assumption was first introduced in reference [24] to simulate environmental perturbations of the development system. In particular, the use of a white noise approach proved useful in simulating environmental shocks, where noise would be increased from small to large values of *σ*. We chose this ansatz due to its analytic simplicity as well as the possibility of studying the effects of noise on a continuous scale. The effects of adding the noise before the action of a canalization function are addressed in the Discussion section.

In this framework, *random binary networks* *W*_*b*_ have elements *w*_*ij*_ with values −1 or 1, assigned with equal probability in our experiments. *Random real-valued networks* *W*_*r*_ have real-valued entries, drawn at random from a standard normal distribution 𝓝(0, 1).

To obtain a *stable binary (real-valued) network*, a *random binary (real-valued) network* *W* and a random initial state *S*(0) are sampled first. Subsequently, *W* is evaluated under (2) (with *ϵ* = 0) and tested for the generation of a stable equilibrium using *S*(0) as initial state. If the *W* does not attain a fixed point (i. e. it attains a limit cycle), both the matrix *W* and initial state *S*(0) are discarded, and a new network and initial state are generated. This procedure is repeated until a network is found for which *S*(∞) is a fixed point.

Stable networks were utilized to emulate a biologically more realistic representation of a population of organisms. In [19], stable networks were mainly considered for tractability reasons, but it is reasonable to assume that in most biological contexts, adult organisms maintain their acquired phenotype. For that reason, we defined stable networks as arising from a pre-selection process, which increases the chance that stable phenotypes are produced. Hence, this process implements an artificial selection for stability of phenotypes at the conclusion of development.

### Normalization function f

For *k* = 2 and *s*_off_ = −1, *f* is the sign function, with sgn(0) = 1. For *s*_off_ = 0, *f* is the Heaviside step function, defined as *H*(*x*) = 1/2(1 + sgn(*x*)), with *H*(0) = 1. For *k* > 2, the sign/Heaviside functions are replaced by a *k*-step function. The function maps values *x* from (−∞, ∞) to values *y* given by Eq (1). The value of *y* is determined by whether *x* lies within particular sections of (−∞, ∞), separated by predetermined boundaries, given by

$$\begin{array}{c} x=-1+\frac{2j}{k},j=1,2,\cdots ,k-1. \end{array}$$ (3)

### Alternative noise models

As an alternative to the simple Gaussian white noise added to the phenotype states, we also explored *random flips* as different implementations of noise. In general, how random flips are implemented requires the specification of three properties: (1) which states a given gene can flip to (transition probabilities), (2) the number of genes that flip, and (3) the timing of the application of noise.

In this study, we consider the effects of two types of flips, *random or no flips* and *neighbor flips*.

**Transition probabilities for flips.** Random flips act on the *i*-th entry of the gene expression state, *s*_*i*_. If *k* = 2, this is just flipping from *s*_on_ to *s*_off_, with probability 0.5. For *k* > 2, there exist three possibilities to implement random flips. First, the random flip could result in any of the possible *k* states (termed *random or no flip* model). Second, it could have any of the other *k* − 1 different states as an outcome (termed *random other flip* model), or third, it could switch to one of the nearest neighbors (the *neighbor flip*, where 0 and 1 only have one nearest neighbor, but intermediate states have two, in which case we chose randomly between the two). We have focused on the *random or no flip* and *neighbor flip* models only.

For example, with *k* = 4 and *s*_*i*_ = 0, the random or no flip model allows switches to any state, including the current state, i. e., {0, 1/3, 2/3, 1} (random or no flip); the random other flip model allows switches to any other state in {1/3, 2/3, 1} with equal probability; and the neighbor flip model only allows *s*_*i*_ = 0 to switch to 1/3 (nearest neighbor).

**Number of gene states flipping.** The strength of noise was varied by deterministically fixing *Q*, the number of genes allowed to flip (*Q* ∈ {0, …, *N*}). The higher *Q*, the more noise phenotypic states were subject to. *Q* was kept constant throughout the simulation. If *Q* was smaller than *N*, the genes affected by noise were chosen at random at each iteration.

**Timing of noise.** Noise can be added to the gene expression vector before or after the matrix multiplication *w*_*ij*_ × *s*_*i*_. If not stated otherwise, we only considered the former case.

### Solving the dynamic equations

Each gene regulatory network subdivides the space of possible phenotypes into disjoint subsets, called *basins of attraction*. These subsets contain equilibria (termed *attractors*) of the dynamical system. Given an initial gene expression state, the dynamical system (2) may converge to an attractor [28, 29], which may be a fixed point or a limit cycle.

**Solving the dynamic equations without noise.** To find equilibria of the dynamics of the developmental model in the absence of noise, we applied cycle detection algorithms on distinct representations of phenotype vectors. All possible *N*-dimensional state vectors *S*(*t*) can be represented by an integer *x* ∈ {0, 1, …, *k* ^*N*^ − 1} using binary or gray code [30], for example.

To assess to which attractor the system (2) converges, we use the number representation of the phenotype state. This allows for an increase in simulation speed of up to 5 times.

Using the integer representation *x*_*i*_ ∈ {0, 1, …, *k* ^*N*^ − 1} for a phenotype state after *i* iterations, we can rewrite Eq (2) as *x*_*i*+1_ = *F*(*x*_*i*_), where *F* is the developmental function. Starting from any initial value *x*_0_, the sequence of iterated values *x*_0_, *x*_1_ = *F*(*x*_0_), *x*_2_ = *F*(*x*_1_), …, *x*_*i*_ = *F*(*x*_*i*−1_),… must eventually generate the same value twice: there must be some *i* ≠ *j* such that *x*_*i*_ = *x*_*j*_. Once this happens, the sequence must continue, repeating the cycle of values from *x*_*i*_ to *x*_*j*−1_.

Whether the system has converged to a solution of Eq (2) can thus be assessed by a cycle detection algorithm. To this end, we used Brent’s algorithm [31] to obtain the orbit’s period (where a period of 1 is a fixed point), the path length to equilibrium, i. e. the time of transition from the initial state to the attractor) and the final state of the system (or the first state of a cycle).

**Solving the dynamic equations with noise.** Since the presence of noise in the developmental model might perturb stable states, we employed a specific criterion to assess the attractors. Under the application of noise, the equilibrium steady state, *S*(∞), is reached when a measure Ψ of the mean deviation from a time average of the evolving phenotype state is smaller than an error threshold *δ* < < 1. Ψ is a functional of the phenotype state:

$$\begin{array}{c} \Psi \left(S\left(t\right)\right)=\frac{1}{\tau }\sum_{t^{\prime }=t-\tau }^{t}d\left(S\left(t^{\prime }\right),\bar{S}\left(t\right)\right) \end{array}$$ (4)

where $\bar{S}(t)$ is the average of states in the time interval (*t* − *τ*, …, *t*) and

$$\begin{array}{c} d\left(S^{a},S^{b}\right)=\frac{1}{N}\sum_{i=1}^{N}{\left(s_{i}^{a}-s_{i}^{b}\right)}^{2} \end{array}$$ (5)

denotes our distance metric between two state vectors *S* ^*a*^ and *S* ^*b*^. If this convergence criterion is not satisfied within T iterations, we assume that there is no steady state. The parameter values employed in the simulations were *T* = 100, *τ* = 10 and *δ* = 10^−4^, following [19, 21]. Previous treatments have shown results to be robust to changes in these parameters [32].

### Sample Size *λ*

The sample size *λ* is the number of network and initial condition pairs (*W*, *S*(0)) sampled and evaluated. For each sample pair employed, the developmental Eq (2) is repeatedly applied to check for the existence of a stable phenotype state *S*(∞). The number of unique phenotypes obtained after *λ* trials is denoted by *U*. The parameter *λ* can be thought of as being determined or bounded by the population size of an organism.

## Results

### Noise is detrimental to phenotype discovery by binary networks with bi-modal gene expression

This section explores whether the expansion of gene regulatory networks to incorporate more traits or genes could represent a viable pathway for an organism to access additional phenotypic states and how noise affects the discovery of such new phenotypes.

We imagine the outcome of the development of an organism to be captured by Eq (2), which in turn depends on the number of phenotype traits *N*, the level of noise *σ*, as well as the randomly chosen initial phenotype *S*(0). Here, we set *k* = 2 (see Methods). To investigate the extent to which such a simple system could access new unique phenotypes, we repeated *λ* = 10^5^ simulations with binary random and stable networks for each *N*.

**Fig 1:**
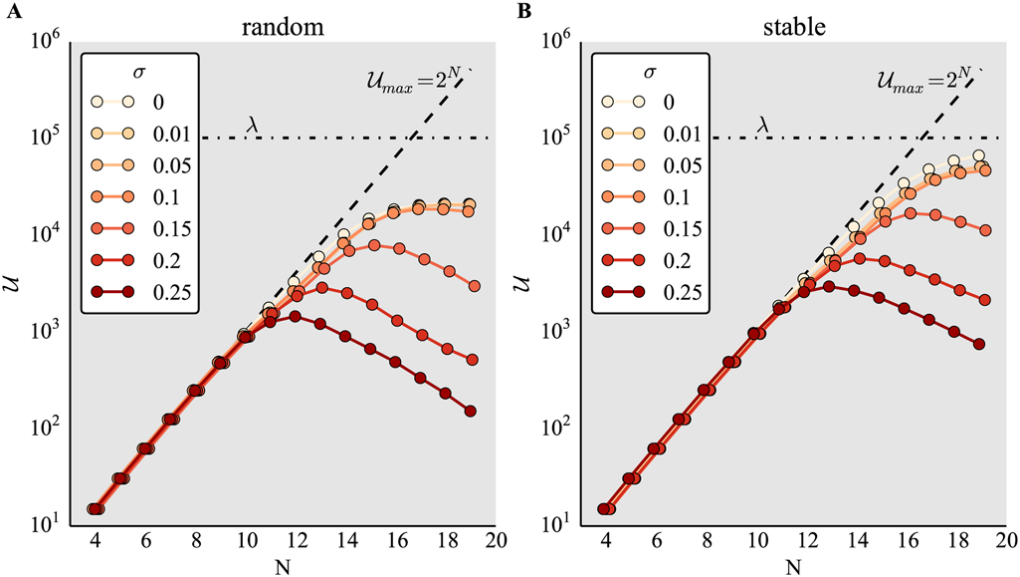
Noise is detrimental to phenotype discovery by binary networks with bimodal gene expression. Number of unique phenotypes *U* accessed for different network size *N* and varying degrees of noise *σ*, for (A) random networks and (B) networks pre-selected for stability. Networks are binary, *W*_*b*_, gene expression is bimodal, *k* = 2, *s*_off_ = 0, maximum of *T* = 100 iterations, and sample size *λ* = 10^5^. Jitter was added to values on the x-axis to avoid overlap of markers. Horizontal dashed lines represent sample size *λ* = 10^5^. Diagonal dashed lines represent the size of phenotype space: 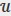
_*max*_ = 2^*N*^.

Fig 1A shows that without the addition of noise, the number of unique phenotypes *U* accessed within *λ* repeats is at its maximum (*k* ^*N*^) for small *N* and random binary networks *W*_*u*_. As *N* increases, the number of unique phenotypic states *U* becomes limited by *λ*, the number of possible developmental trajectories examined. For large *N*, the number of discovered phenotypes *U* saturates at a value below the sampling size *λ*. The larger the noise in the system, the larger the decrease in the number of accessible phenotypes *U* at saturation.

Fig 1B shows an analogous situation with stable regulatory networks *W*_*s*_. The general pattern here is similar to that in Fig 1A, but with more phenotypic states *U* discovered at all noise levels, and a saturation level of discovered phenotypes close to the maximum *λ*.

To assess the robustness of these results, we investigated whether the main features of of the decline of the attained *U* with increasing noise levels were preserved for different implementations of noise. To this end, we tested different implementations of noise for increasing numbers of traits *N* and fixed *k*.

**Fig 2:**
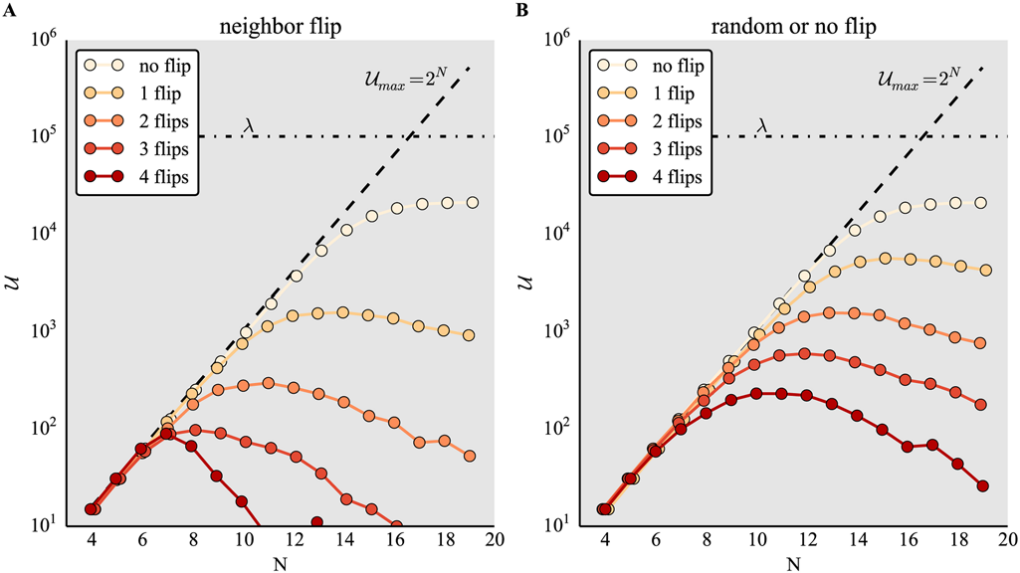
Effect of implementations of noise on phenotype discovery for *k* = 2. Number of unique phenotypes *U* accessed for different network sizes *N* and varying number of gene flips for noise models (A) neighbor flip and (B) random or no flip. Networks are random (not pre-selected for stability). Other parameters and dashed lines are as in Fig 1.

Fig 2A shows how neighbor flip noise (see Methods) affects the discovery of the number of unique phenotypes *U* by random binary networks of increasing *N*. Here, we set *λ* = 10^7^ and *k* = 2. The results are qualitatively similar to those in Fig 1: all possible phenotypes are discovered if *k* ^*N*^ is small compared to *λ*, and saturation ensues if *N* increases. Higher noise levels decrease phenotype accessibility. Fig 2B shows the same qualitative result for a similar implementation of noise, the random or no flip (see Methods). Noise is effectively smaller for the latter model, because on average, genes selected to flip are only flipped half of the time (*random or no flip*). For the former, genes selected to flip always flip (*neighbor flip*).

Surprisingly, these results suggest that for large enough sample sizes, random binary networks seem capable of accessing all potential phenotypes if the genome size is increased. Access to all potential phenotypes is facilitated by the large number of available and different binary networks, which scales as 2^*N*×*N*^, whereas the number of potential phenotypes is *U*_max_ = 2^*N*^. Hence, despite the degeneracy of several different networks mapping initial phenotypes onto the same stable phenotype, it is possible to access all possible phenotypes if enough random networks are sampled. This behavior extends the observations made in [4], under a model that involves a single development step, as well as the model here presented. With increasing *N*, the number of all possible phenotypes starts to surpass the sample size *λ*, which limits how many phenotypes can potentially be discovered. Hence, the fraction of accessible phenotypes must decline with *N*, as the maximum number of distinct phenotypes (the size of the phenotype space), *U*_max_ = 2^*N*^, approaches *λ*. This explains why in reference [4] only a fraction of all phenotypes were visible to the developmental process at a fixed *λ* = 10^4^.

Noise is exclusively detrimental to phenotype discovery whenever the maximum number of possible phenotypes approaches the sampling size *λ*. This effect is qualitatively robust to different implementations of noise, in accord with [26]. The pre-selection of regulatory networks *W*_*s*_ leads to substantially greater phenotype discovery close to the theoretical maximum *λ*. This is probably due to the effective increase in the number of explored simulations due to the pre-selection of *W*_*s*_ networks.

### The enhancement of phenotypes accessibility by noise in binary networks depends on *λ*

In some instances, the expansion of regulatory networks by the acquisition of additional genes might be biologically constrained. The major mechanism by which regulatory networks can increase in size is by gene duplication [33, 34] and co-option of other genes [35, 36]. However, increasing the network size could be biochemically unfeasible or entail prohibitive costs to the organism. After a gene duplication event, for example, the new copy stands little chance of rising to fixation and being retained long-term (perhaps through acquiring new functions or subdividing old ones), if it is not expressed [37]. But being expressed alongside the old copy implies increased dosage, which is frequently deleterious [38].

To address this issue, we examined alternative means of network expansion. One way to increase the size of phenotypic space without altering the number of genes *N* in the network, is to increase the number of intermediate gene expression states *k*. With *N* fixed, the phenotypic space then increases polynomially with *k* as *k* ^*N*^.

Setting *k* > 2 constitutes a departure from commonly employed models, where only two expression states have been considered [19, 32]. However, this assumption does have a biological basis. For example, during embryonic development and many cellular processes, there are threshold responses and gradient-driven processes, which entail that genes have multiple expression states [39].

**Fig 3:**
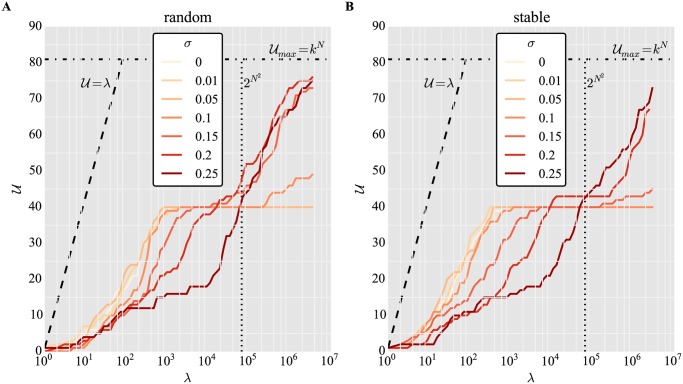
Noise can increase the number of phenotypes accessible by binary networks in high sampling regimes. Number of unique phenotypes *U* accessed for different sampling sizes *λ* and varying degrees of noise *σ*, for (A) random networks and (B) networks pre-selected for stability. Networks are binary, *W*_*b*_, network size *N* = 4, number of gene expression states *k* = 3, *s*_off_ = 0, maximum of *T* = 100 iterations. Horizontal dashed lines represent the size of phenotype space, 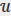
_*max*_ = *k* ^*N*^ = 81. Vertical dashed lines represent the size of genotype space, 2^*N*^2^^ = 65536. Diagonal dashed lines represent discovery rate without redundancy and overlap, where every new sample yields a new phenotype, i. e. 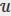 = *λ*.

Fig 3A shows the number of unique gene expression fixed points *U* attained by the developmental model (2) with random binary networks *W*_*u*_ for different sample sizes *λ* and varying degrees of Gaussian noise *σ*. The number of genes is fixed at *N* = 4, and the number of expression states per trait is *k* = 3. For small *λ*, noise decreases the number of unique phenotypes accessed. This situation reverses at larger *λ*, where noise enables more unique phenotypes to be accessed. An analogous situation is observed in Fig 3B for stable binary matrices *W*_*s*_, where the increase due to noise is less pronounced.

These figures capture the degeneracy in unique stable phenotypes induced by many distinct regulatory network matrices *W*. Since many *W*’s define identical equilibrium phenotypic states in the absence of noise, sampling only a small fraction (small *λ*) of them with replacement will lead to an overrepresentation of the most degenerate phenotypes. As the sample size increases, additional phenotypes of smaller degeneracy are discovered, leading to a speed-up in the accessibility of phenotypes. As *λ* surpasses the number of all possible binary matrices of size *N* = 4, 2^*N*×*N*^ = 65, 536, saturation starts to occur in *U*. It is at this transition that noise becomes advantageous, making previously inaccessible phenotypes appear.

### Noise can moderately increase the number of phenotypes accessible by binary networks for intermediate k and specific implementations of noise

Having found that noise can enhance the discovery of phenotypes for large sample sizes, we investigated whether these effects persist for *k* > 3. To this end, we measured *U* for a fixed sample size *λ*, a fixed number of genes *N*, but varying *k* and noise levels *σ*.

**Fig 4:**
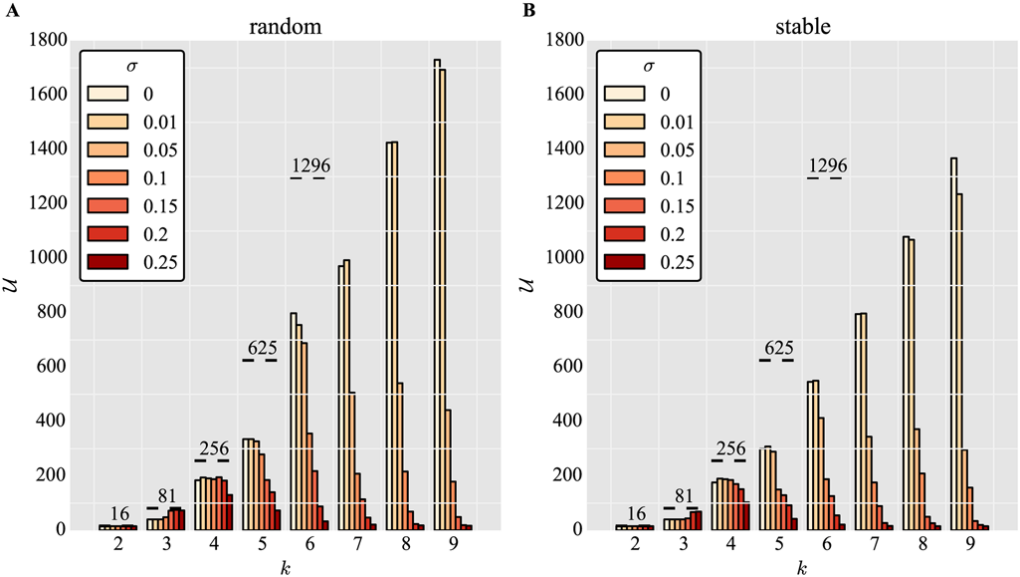
Noise can moderately increase the number of phenotypes accessible by binary networks for intermediate *k*. Number of unique phenotypes *U* accessed for different numbers of gene expression states *k* and varying degrees of noise *σ*, for (A) random networks and (B) networks pre-selected for stability. Networks are binary, *W*_*b*_, network size *N* = 4, *s*_off_ = 0, maximum of *T* = 100 iterations, and sample size *λ* = 2.4 × 10^6^. Horizontal dashed lines represent the size of phenotype space: 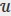
_*max*_ = *k* ^4^.

Fig 4A shows the effects of noise and the number of states per trait on phenotype accessibility for *λ* = 2.4 × 10^6^, *N* = 4 and randomly chosen networks *W*_*u*_. In contrast to the increase with network size shown in Fig 1, only a fraction of the possible states are accessible to binary matrices by the end of the development phase. As *k* increases, the fraction of accessible phenotypes tends to decrease on average (in accord with [4]). Noise only very moderately increases *U* for *k* = 3, 4, 7, but mainly decreases *U* after *k* > 4.

Fig 4B shows the analogous situation for stable networks. Here, noise has a similar, slightly beneficial effect for *k* = 3, 4, 5, 6 and for low levels of noise *σ* = 0.05. Overall, almost all levels of noise diminish *U*. As previously seen in Fig 3, noise leads to less phenotype discovery in stable networks compared to random networks.

**Fig 5:**
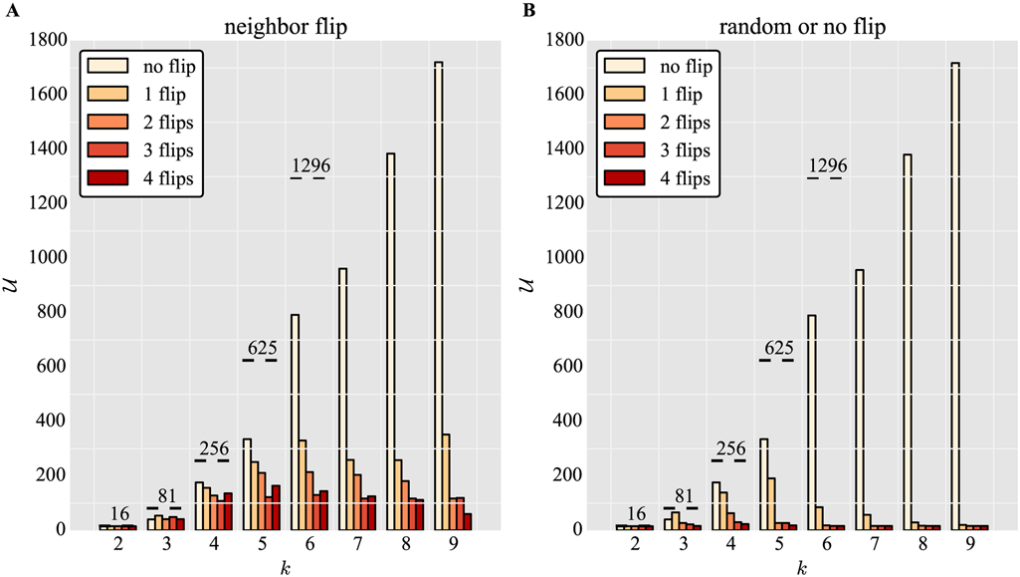
Effect of implementations of noise on phenotype discovery for varying *k* and *N* = 4. Number of unique phenotypes *U* accessed for different different numbers of gene expression states *k*, and varying number of gene flips for noise models (A) neighbor flip and (B) random or no flip. Networks are random (not pre-selected for stability). Other parameters and dashed lines are as in Fig 4.

We then asked how distinct implementations of noise affect unique phenotype discovery for fixed *N* and variable *k*. Fig 5A shows that neighbor flip noise reduces the discovery of unique phenotypes at all levels. As expected, the results are more pronounced for random flip noise, shown in Fig 5B. Here, noise substantially diminishes the number of phenotypes discovered at all levels and with growing *k*.

This shows that the results on phenotype discovery are robust to how noise is implemented for *k* = 3, but divergent behaviors emerge depending on the type of noise for *k* > 3.

### Noise decreases the number of phenotypes accessible by real-valued networks

Much of our knowledge about biological networks comes in the form of binary information about the activation or repression of some form of expression (using microarray data, for example [40]). This is a consequence of simplification: it is much easier to describe a regulatory interaction in qualitative terms than to quantify it by interaction strengths and binding affinities [41, 42]. Consequently, most models use either binary weights or random numbers at the start of evolutionary simulations [19, 25]. Another reason for modeling regulatory interactions as binary is that fine-tuning of interaction strengths can prove costly or even impossible for an organism, especially in the presence of cellular noise [42–44].

It is the relative size of these interaction strengths however, that is relevant to the dynamics represented by Eq (2) [19, 45]. Despite its costs, organisms could evolve fine-tuning of some interactions if it were beneficial in the long term. Such interactions can be captured by the use of real-valued networks, instead of binary networks.

**Fig 6:**
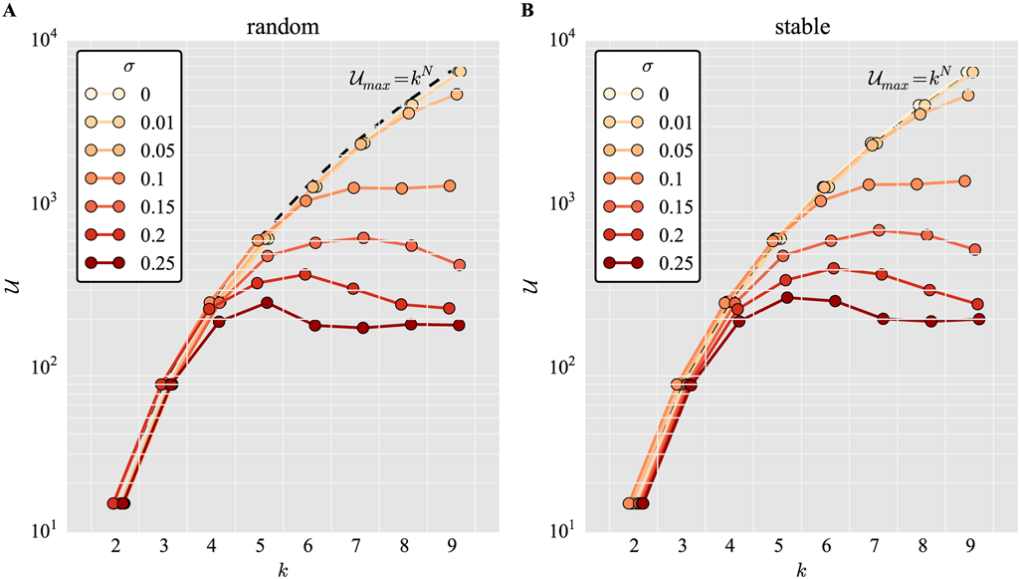
Noise decreases the number of phenotypes accessible by real-valued networks. Number of unique phenotypes *U* accessed for different numbers of gene expression states *k* and varying degrees of noise *σ*, for (A) random networks and (B) networks pre-selected for stability. Networks are real-valued, *W*_*r*_, network size *N* = 4, *s*_off_ = 0, maximum of *T* = 100 iterations, and sample size *λ* = 1.7 × 10^6^. Jitter was added to values on the x-axis to avoid overlap of markers. Dashed lines represent the size of phenotype space: 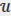
_*max*_ = *k* ^4^.

Fig 6A shows that random real-valued networks, developing as specified in (2) (see Methods), can access almost all possible unique phenotypes *U* in the absence of noise. Here, we fixed *λ* = 1.6 × 10^6^ and *N* = 4 for all simulations. Noise has a detrimental effect as *k* increases. Fig 6B shows the effect of noise on stable real-valued networks. The effects of noise are qualitatively and quantitatively similar in Fig 6A and 6B.

These results indicate that fine tuning the regulatory interactions on genes by using real-valued networks could allow a population of organisms to access substantially more phenotypes than binary networks. This effect is much stronger than the incremental improvement on phenotype discovery conferred by pre-selecting real-valued networks for stability.

## Discussion

In this study, we have explored two main ways to increase the absolute number of phenotypes accessible through developmental processes, and in addition, the role of noise in phenotype discovery. First, by increasing the number of genes (*N*), we found that in the absence of noise, all phenotypes become accessible given large enough sample sizes. Second, with an increase in the number of gene expression states (*k*), noise can moderately increase phenotype discovery for small *k*. These results are valid for both, random and stable binary networks.

When the number of gene expression states *k*, becomes larger than two, sample sizes and noise interact in a complex fashion to influence the accessibility of new unique phenotypes. In particular, for *k* = 3, the larger the sample size, the larger the noise levels required to obtain a marginal increase to phenotype discovery. This suggests that increasing the number of gene expression states could open the way to novel phenotypes for organisms whose development is subject to strong noise.

The improvement in phenotype accessibility with increasing noise is most pronounced for *k* = 3, where almost double the number of unique phenotypes can be accessed under high noise compared with no noise. This beneficial effect of noise at large sampling sizes erodes fast with larger *k*. The biological importance of noise as a facilitator of phenotype discovery is therefore limited to small *k* values, where the absolute increase in newly accessed phenotypes is small in absolute terms, but large in relative terms. The diversification of gene expression states from only “on” and “off” to more expression states could therefore be halted before quasi-continuous expression states are attained. However, a more detailed investigation of this question would require accounting for the cost to an organism of acquiring a genetic machinery capable of expressing multiple states of gene expression.

We also explored the behavior of networks that allow for fine-tuning of regulatory interactions, namely real-valued networks. We found that compared to binary networks, real-valued networks substantially increase access to phenotypes for *k* > 2. Again, noise was exclusively detrimental for phenotype accessibility.

Finally, we also studied distinct implementations of noise to assess the robustness of our results. We found that the behavior of the developmental model did not change qualitatively with noise types.

In previous studies, a more differentiated model was employed to represent development, where genotypes were equated with transcription factors for expression, and phenotypes were equated with the gene expression itself [4]. In this context, development entails the one-time application of a mapping function on a genotype to obtain an end-state phenotype.

With one-iteration development, an increase in the size of a network or a regulatory system, also increases the number of potential phenotypes accessible to the system. However, not all potential phenotypes are accessed starting from random genotypes in random binary networks [4]. Redundancy or degeneracy effects [3, 46, 47] and, more generally, the nonlinearity of the genotype-phenotype map, render most of the phenotypic space inaccessible to the regulatory system [5]. This severely limits the space of possibilities accessible by evolutionary search [4].

A caveat of this study might lie in the sacrifice of the distinction between transcription factor expression and gene expression –as adopted in [4]– in favor of a simpler interpretation of the developmental process; in our approach, genes act as transcription factors themselves. In this paper, we have studied how phenotypes stabilize after the repeated application of the mapping function. This reflects a reinterpretation of the developmental system: Development starts at an initial phenotype, which is iteratively mapped onto next-generation phenotypes by the developmental mapping function until either a stable phenotype is reached or another behavior becomes apparent. In this perspective, genotypes correspond to the regulatory network acting on the phenotypes, whereas the gene expression states are the phenotypes.

To address the issue of biological constraints imposed by the costs of the acquisition of additional genes in gene regulatory networks, we departed from commonly employed models and introduced the assumption of multiple discrete gene expression states, *k* > 2. Although it is difficult to estimate how widespread multimodal gene expression is in nature, the increasing accumulation of gene expression data should lead to further elucidation of this issue. The assumption is in accordance with evidence for the existence of multiple expression states, including *k* = 3, that have recently been inferred from micro-RNA data by bioinformatic methods [48]. However, the full extent to which such states are inter-dependent in natural gene regulatory networks is unclear. Inter-dependent gene expression would markedly affect phenotype exploration for the organisms, potentially aggravating the effect of degeneracy.

Our adoption of the stability criterion based on a variance measure Ψ as well as restrictive parameter values, precludes the study specific dynamics. In particular, limit cycle behavior, or the alternating adoption of two distinct phenotypes in an organism, termed phenotypic switching, are not captured by this measure. For the values of *δ* employed in this study, these types of dynamics are not identified as stable phenotypes.

As a consequence, we have neglected a known and important stabilizing role of noise for the maintenance of alternating phenotypes [49] by focusing on a restricted set of unchanging stable states. To allow for changing, but non-random phenotypes would require the implementation of more sophisticated measures of phenotype stability, as well as an implementation of noise that acts after the matrix multiplication in Eq (2), and not before. Such measures would have to account for the cycling nature of phenotype dynamics and allow contributions from randomness to the dynamics to be disentangled from recurring regularities. Studies that focus on how reversible phenotypic switching is induced by noise also employ more detailed models of gene regulatory interactions [49].

A more realistic representation of a population of organisms of the same species might restrict the space of possible regulatory networks to a small fraction of all possible networks. To address this issue, we investigated stable networks. The pre-selection procedure involved in stable network choices mimicked artificial selection, reducing the space of networks investigated.

We also chose to simulate development starting from random initial phenotypes. This assumption is probably unrealistic. However, our focus was on the overall effect of noise on phenotype discovery. To satisfy more realistic assumptions, a decision needs to be made on which sub-space of phenotypes corresponds to more realistic biological phenotypes. Since this is currently unknown, such a restriction would be the same as studying random initial conditions.

To assess whether phenotypes had reached an equilibrium state under the influence of noise, we required that a variance-like statistic should satisfy a stability condition after *T* iterations of the developmental mapping function. The value of *T* was fixed for all simulations, and larger *T* might have led to more beneficial effects of noise, since more iterations would have been available for phenotypes to reach stable end states (see S1 Fig and S2 Fig). *T* was held constant to mimic the limited lifetime of the developing organism.

Our results should be interpreted in the context of whether stochasticity can increase phenotypic variation in organisms. By generating phenotypic heterogeneity, stochasticity is expected to be particularly beneficial to microbial cells that may need to adapt efficiently to sudden changes in environmental conditions [13]. In eukaryotes such as yeast, it has recently been suggested that, for genes such as plasma-membrane transporters, noise in elevated expression is advantageous, is subject to positive selection, and facilitates evolution of adaptive gene expression [50]. This suggests that in specific regimes, stochastic models can evolve solutions where purely deterministic models fail [51].

We suggest that for noise to be beneficial, in the sense of an increase in the number of accessible phenotypes of binary systems, very specific conditions need to be satisfied. If increasing the number of genes in a network or fine-tuning the binding affinities of specific transcription factors to regulatory regions were costly to the organism, noise inherent in the process of gene regulation could be useful in increasing the number of accessible phenotypes.

These considerations also shed new light on the relative importance of regulatory evolution versus gene evolution, studied in the context of the evo-devo paradigm [52–54]. Phenotypes are made accessible to or concealed from the system undergoing development through changes in the current phenotypic state instead of in other “structural” processes such as gene duplication or mutation. This suggests an additional role for regulatory evolution in promoting or supporting evolutionary novelties [55].

## Supporting Information

S1 Fig Increasing T increases the number of phenotypes accessible, but the effect size is small. *k* = 2. Number of unique phenotypes *U* accessed for different sampling sizes *λ* and increasing developmental time T, for increasing degrees of noise *σ*. Other parameters are as in Fig 3.
(PDF)

S2 Fig Increasing T increases the number of phenotypes accessible, but the effect size is small. *k* = 7. Number of unique phenotypes *U* accessed for different sampling sizes *λ* and increasing developmental time T, for increasing degrees of noise *σ*. Other parameters are as in Fig 3.
(PDF)
