# Supplementary figures and images for: Phenotype Accessibility and Noise in Random Threshold Gene Regulatory Networks
Source: PLoS One. 2015 Apr 28;10(4):e0119972. doi: 10.1371/journal.pone.0119972 (PMC4412837; doi:10.1371/journal.pone.0119972)

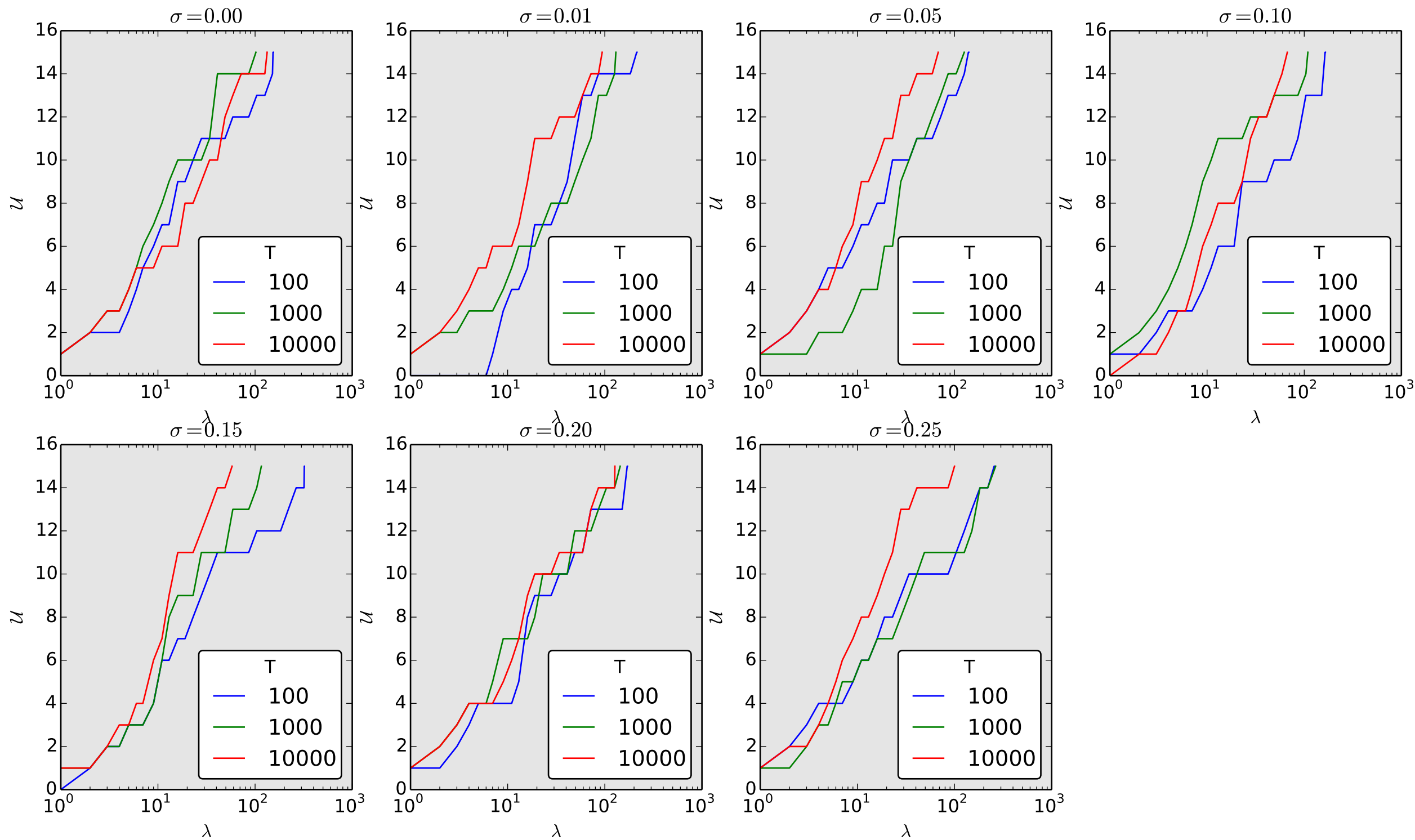

Supplement: S1 Fig — Number of unique phenotypes U accessed for different sampling sizes λ and increasing developmental time T, for increasing degrees of noise σ. Other parameters are as in Fig 3. (PDF) [file pone.0119972.s001.pdf]

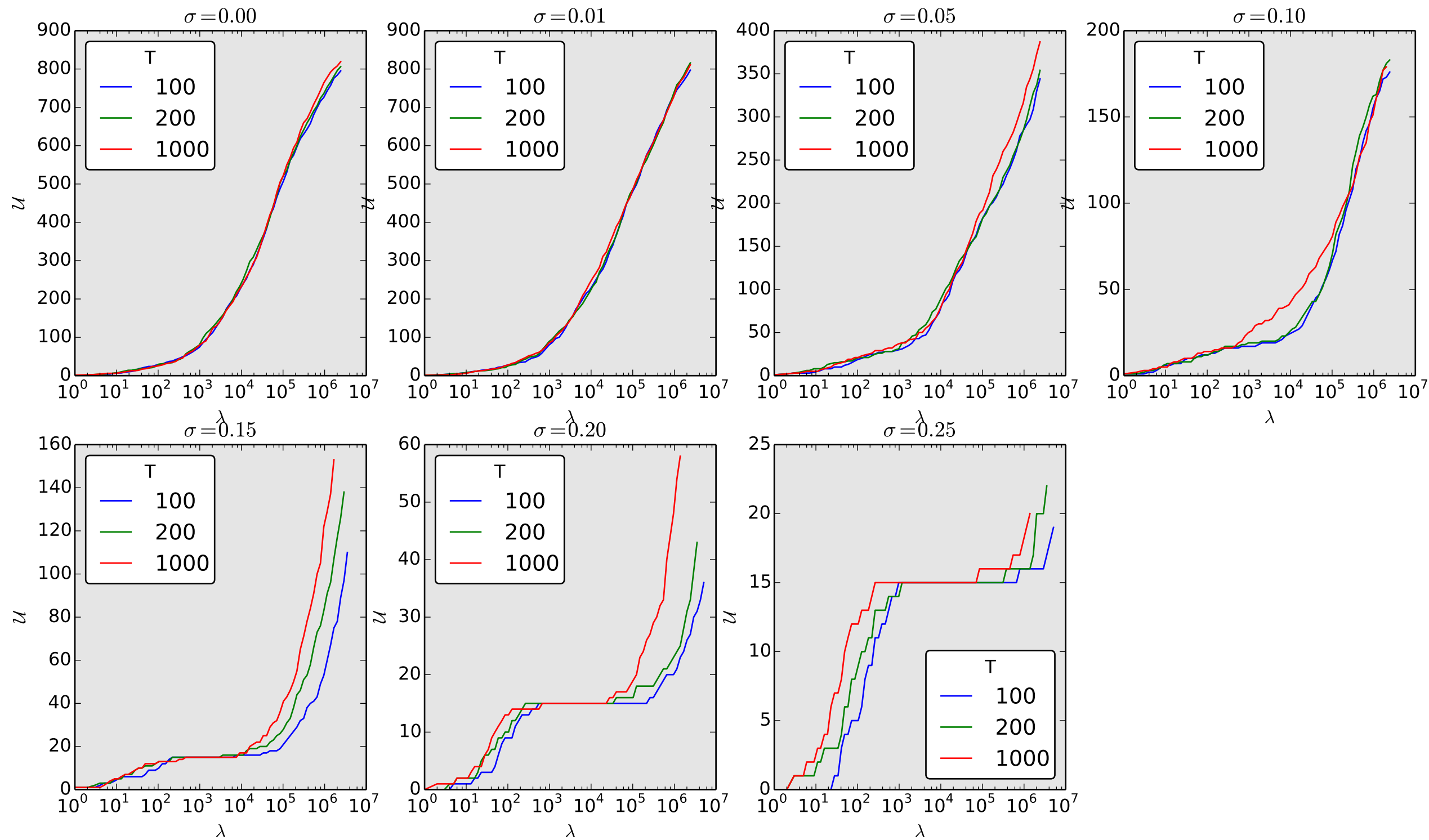

Supplement: S2 Fig — Number of unique phenotypes U accessed for different sampling sizes λ and increasing developmental time T, for increasing degrees of noise σ. Other parameters are as in Fig 3. (PDF) [file pone.0119972.s002.pdf]
